# The angular structure of ONC201, a TRAIL pathway-inducing compound, determines its potent anti-cancer activity

**DOI:** 10.18632/oncotarget.2890

**Published:** 2015-01-13

**Authors:** Jessica Wagner, Christina Leah Kline, Richard S. Pottorf, Bhaskara Rao Nallaganchu, Gary L. Olson, David T. Dicker, Joshua E. Allen, Wafik S. El-Deiry

**Affiliations:** ^1^ Hematology/Oncology Division and Penn State Hershey Cancer Institute, Penn State University, Hershey, PA, USA; ^2^ Laboratory of Translational Oncology and Experimental Cancer Therapeutics, Department of Medical Oncology and Molecular Therapeutics Program, Fox Chase Cancer Center, Philadelphia, PA, USA; ^3^ Provid Pharmaceuticals, Inc., Monmouth Junction, NJ, USA; ^4^ Oncoceutics, Inc., Hummelstown, PA, USA

**Keywords:** ONC201, TIC10, cancer, TRAIL pathway, Foxo3a, Akt, ERK

## Abstract

We previously identified TRAIL-inducing compound 10 (TIC10), also known as NSC350625 or ONC201, from a NCI chemical library screen as a small molecule that has potent anti-tumor efficacy and a benign safety profile in preclinical cancer models. The chemical structure that was originally published by Stahle, et. al. in the patent literature was described as an imidazo[1,2-a]pyrido[4,3-d]pyrimidine derivative. The NCI and others generally accepted this as the correct structure, which was consistent with the mass spectrometry analysis outlined in the publication by Allen et. al. that first reported the molecule's anticancer properties. A recent publication demonstrated that the chemical structure of ONC201 material from the NCI is an angular [3,4-e] isomer of the originally disclosed, linear [4,3-d] structure. Here we confirm by NMR and X-ray structural analysis of the dihydrochloride salt form that the ONC201 material produced by Oncoceutics is the angular [3,4-e] structure and not the linear structure originally depicted in the patent literature and by the NCI. Similarly, in accordance with our biological evaluation, the previously disclosed anti-cancer activity is associated with the angular structure and not the linear isomer. Together these studies confirm that ONC201, produced by Oncoceutics or obtained from the NCI, possesses an angular [3,4-e] structure that represents the highly active anti-cancer compound utilized in prior preclinical studies and now entering clinical trials in advanced cancers.

## INTRODUCTION

The endogenous anti-tumor protein TRAIL (tumor necrosis factor-related apoptosis-inducing ligand) was previously investigated as a therapeutic target in oncology due to its ability to activate death receptors in tumor cells, leading to apoptosis [1, 2]. Despite this ability to selectively induce apoptosis in a variety of tumor and transformed cells without affecting normal cells, TRAIL was perceived to have notable shortcomings as a short-lived protein therapeutic that limited interest in its clinical development [1, 3]. Based on its ability to induce the TRAIL gene, we previously obtained and studied the compound NSC-350625 from the National Cancer Institute (NCI) Diversity Set II, which the NCI depicted as 7-benzyl-10-(2-methylbenzyl)-2,6,7,8,9,10-hexahydroimidazo[1,2-a]pyrido[4,3-d]pyrimidin-5(3H)-one [4]. This compound, which is referred to now as ONC201 by Oncoceutics, Inc. that is developing the molecule as an anticancer drug, was originally synthesized and described in a patent application published in 1973 without disclosure of biological data [5].

Preclinical studies revealed that ONC201 has robust anti-tumor effects by activating the TRAIL pathway through an upstream dual blockade of Akt and ERK that activates Foxo3a to transcriptionally up-regulate the TRAIL gene [4, 6, 7]. ONC201 possesses numerous ideal drug properties, including a broad spectrum of activity, wide safety margin, robust stability, aqueous solubility, favorable pharmacokinetics, and oral activity; all of which are superior to TRAIL-based therapies and have prompted clinical development of the compound [4].

We noticed a striking superiority for the preclinical anticancer activity of ONC201 obtained from the NCI and Oncoceutics compared to a compound obtained from a commercial source that synthesized the molecule through an alternative synthetic route. In parallel to investigating this disparity, Jacob *et al*. recently reported a series of NMR and X-ray crystallography studies that determined the chemical structure of ONC201/NSC350625 obtained from the NCI to be an angular [3,4-e] isomer of the structure reported in the original disclosure by Stahle et al. and subsequent disclosures of the molecule by the NCI and Allen *et al* [8]. This angular [3,4-e] isomer has the imidazo ring fused at an angle to the axis of the pyridopyrimidine rings.

Considering this clarification of the angular isomeric structure of ONC201, we performed structure elucidation studies with ONC201 produced by Oncoceutics. These studies extended beyond the purview of mass spectrometry to include ^1^H and ^13^C NMR spectroscopy, IR spectroscopy, and X-ray crystallography. The results of these studies definitively establish the structure of ONC201 dihydrochloride salt material manufactured by Oncoceutics for preclinical and clinical use as the angular [3,4-e] isomer (i.e., 7-benzyl-4-(2-methylbenzyl)-2,4,6,7,8,9 -hexahydroimidazo[1,2-*a*] pyrido[3,4-*e*] pyrimidin-5(1*H*)-one) and that this is the same structure as the material distributed by the NCI as a free base. Further studies comparing ONC201 with a sample of the [4,3-d] linear isomer revealed that these two isomers possess strikingly disparate biological activity, with only ONC201 demonstrating therapeutic potential.

## RESULTS

### Determination of ONC201 chemical structure by NMR and X-ray crystallography

**Figure 1:**
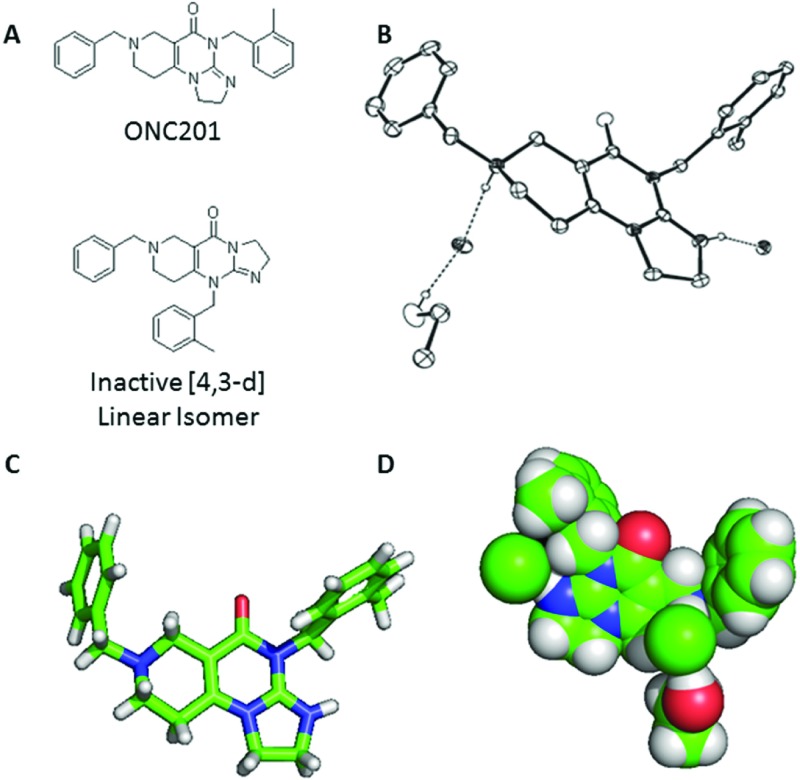
X-Ray crystal structure of ONC201 **(A)** Molecular structure of ONC201 and the [4,3-d] linear isomer. **(B)** Representation of the molecular structure of ONC201 dihydrochloride as determined by X-ray crystallography. **(C)** Ball and stick model and **(D)** space-filling model of ONC201 based on X-ray crystallography data.

**Figure 2:**
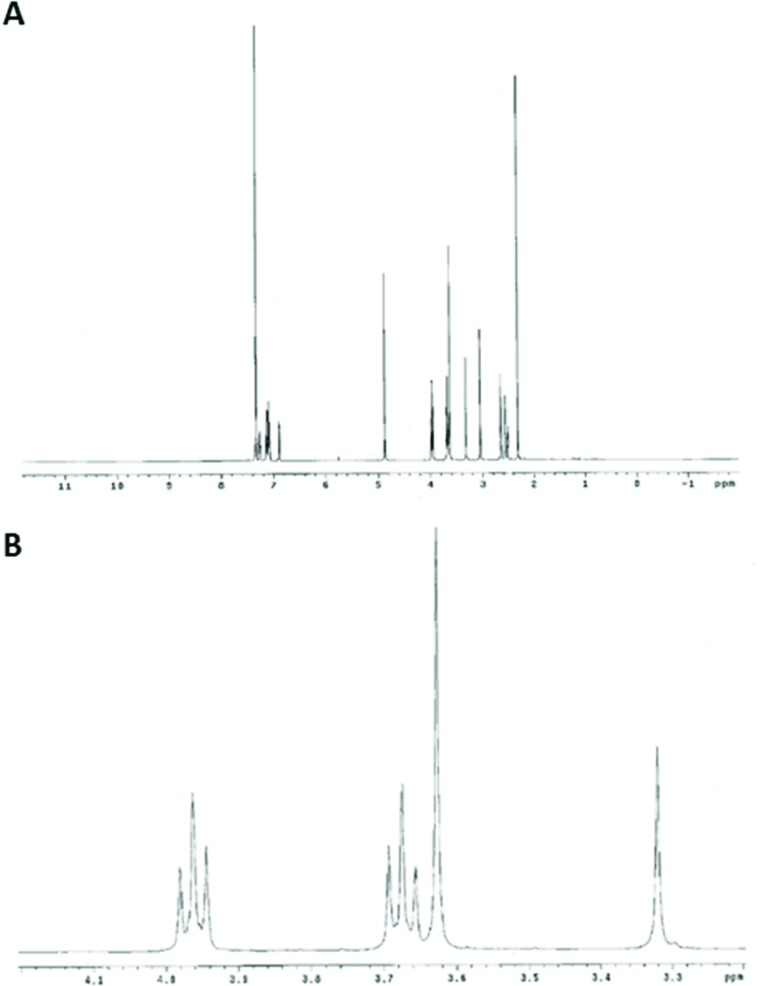
Elucidation of ONC201 structure by NMR **(A)** ^1^H and **(B)** ^13^C NMR Spectrum of ONC201 in d_6_-DMSO. Peak assignments provided in Table 1.

After the preclinical studies of ONC201/NSC350625 revealed its robust anti-tumor effects by activating the TRAIL pathway, we began to observe a disparity between the Oncoceutics and NCI obtained ONC201 material and material obtained from an alternatively synthesized commercial source. During this investigation, a recent publication reported a structure for ONC201 obtained from the NCI that is an angular [3,4-e] isomer of the linear structure depicted in previous publications (Figure 1A) [8]. To confirm the molecular structure of ONC201 manufactured by Oncoceutics for preclinical and clinical use, we performed 1D and 2D NMR analyses of ONC201, in solution with d_6_-DMSO as the solvent (Figure 2; Figure S1–S3). These conditions produced NMR data that allowed for full chemical shift assignments corresponding to the angular [3,4-e] structure of ONC201 (Table 1). The assignment strategy consisted of integration of the 1D ^1^H spectrum that yielded the expected number of protons in appropriate chemical shift ranges.

**Table 1:** Chemical shifts for ONC201 ^1^H and ^13^C NMR analysis in d6-DMSO at 25°C Positions are defined in the chemical structure to the right

Position	1H	13C	
2	--	146.97	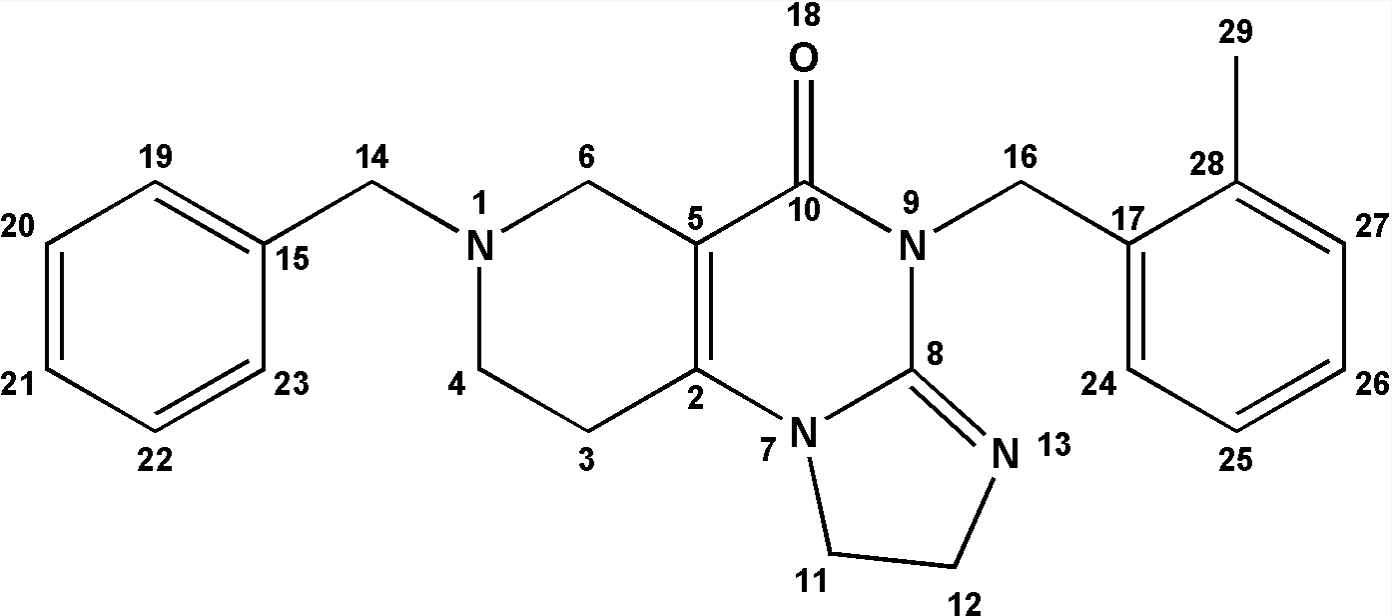
4	2.56/2.56	25.61	
5	2.65/2.65	48.14	
6	--	99.11	
7	3.05/3.05	48.41	
8	--	151.85	
10	--	160.83	
11	3.96/3.96	46.26	
12	3.68/3.68	49.88	
14	3.63/3.63	61.05	
15	--	138.04	
16	4.87/4.87	41.98	
17	--	134.74	
19	7.33	128.54	
20	7.33	129.00	
21	7.26	127.41	
22	7.33	129.00	
23	7.33	128.54	
24	6.89	125.07	
25	7.10	126.46	
26	7.09	126.14	
27	7.13	129.46	
28	--	134.74	
29	2.31	18.38	

The ROESY data indicated the proximity of the methyl group 29 to its neighboring aromatic proton 27, and to the bridging benzylic methylene 16. Also indicated was the proximity of the other methylene bridge 14 to the ortho protons 19 and 23 of the aromatic ring, and to the 4 and 6 protons of the naphthyridine component. Consistent with this, ROEs were observed across the bridge, between 19, 23 and 4,6. The through-bond connectivity of the imidazoline protons 11 and 12 was revealed in the DQCOSY spectrum (not shown). The remaining non-aromatic group 3 was assigned by default, as its connectivity to 4 in the DQCOSY was obscured by the diagonal. Similar results consistent with the angular structure were obtained in CDCl_3_ solvent as well with ONC201.

Next, we conducted X-ray crystallography studies with ONC201 dihydrochloride to definitively elucidate its molecular structure. Optimal crystallization was obtained with ethanol and a trace of water. Under these conditions, ONC201 dihydrochloride crystallizes in the monoclinic space group P21/c (systematic absences 0k0: k=odd and h0l: l=odd) with *a* = 11.7746(3)Å, *b* = 10.3998(2)Å, *c* = 21.9517(5)Å, β=95.1950(10)°, *V* = 2677.02(10)Å3, *Z* = 4, and dcal*c* = 1.254 g/cm^3^. In agreement with the NMR analysis, the structure derived from this X-ray crystallography study confirmed the angular [3,4-e] structure of ONC201 (Figure 1B–1D; Figure S4, Table S1–S6).

### ONC201 and the [4,3-d] linear isomer are indistinguishable by mass spectrometry

**Figure 3:**
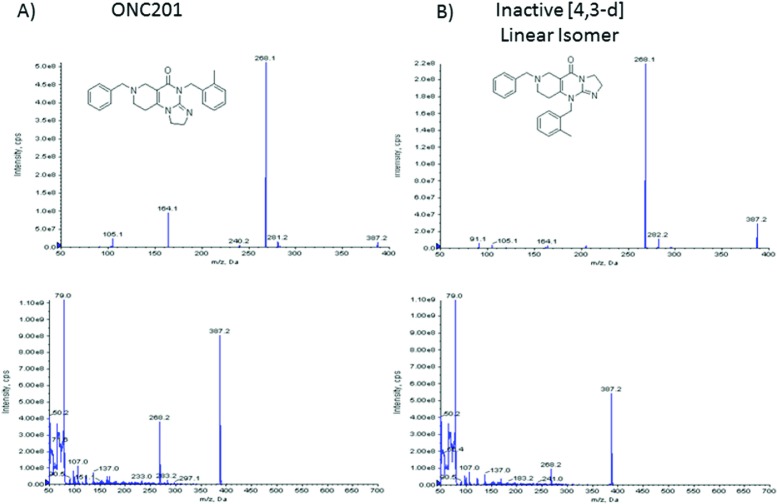
ONC201 and its linear isomer are indistinguishable by mass spectrometry **(A)** Mass spectrometry analysis of ONC201 and **(B)** the [4,3-d] linear isomer.

To compare the spectroscopic profiles of ONC201 and the [4,3-d] linear isomer, we performed mass spectrometry analysis of the two compounds (Figure 3A–3B). Both ONC201 and the [4,3-d] linear isomer exhibited a 387.2 m/z mass spectrum with only subtle differences that were deemed insignificant. Both compounds yielded large, characteristic fragment ions at 79.0, 260.2, and 387.2 m/z, indicating identical fragmentation between the two molecules. Additional ionic fragments were present at lower concentrations with similar distributions from both small molecules. These results are in agreement with the mass spectra previously reported by Allen *et al*. and demonstrate the inability to distinguish ONC201 from the linear isomer by fragmentation patterns in mass spectrometry.

### ONC201 and the [4,3-d] linear isomer have distinct NMR but similar IR spectra

**Figure 4:**
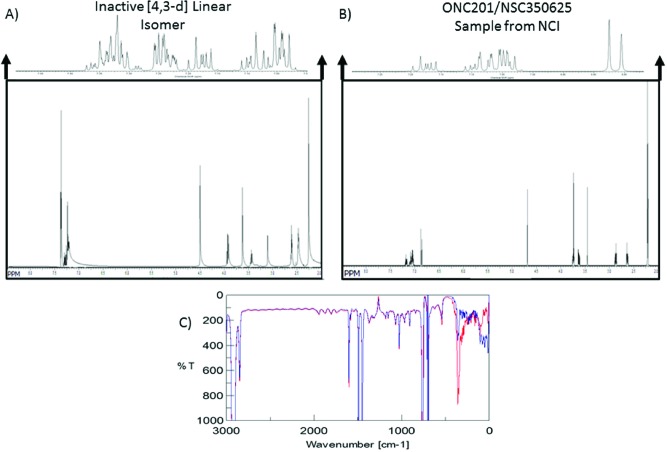
ONC201 and its linear isomer can be distinguished by subtle spectroscopic differences **(A)** NMR spectra of the [4,3-d] linear isomer and **(B)** NMR Spectra for ONC201 obtained from the NCI. Spectra were analyzed using HMBC and MestReNova. **(C)** IR spectra of linear isomer (blue) ONC201 (red) in dichloromethane.

NMR analysis revealed that the small distance between the carbonyl carbon atom and the imidazole rings in the linear isomer allow the aromatic peaks to appear to overlap within 7.2–7.4 ppm, whereas the peaks from ONC201 are slightly farther apart within that range (Figure 2, Figure 4A). Other minor proton shift differences between the two structures are also evident. Three peaks between 3.4–4.0 ppm are congested within the spectra of the ONC201; however, the linear isomer shows two clear triplet peaks as reported in the publication by Jacob, et al. (Figure 4A–4B). Notably, ONC201/NSC350625 (free base) obtained from the NCI, and used in the discovery and initial pharmacology studies of ONC201, showed a very similar NMR spectrum to the ONC201 clinical dihydrochloride material manufactured by Oncoceutics. Thus NMR analysis is able to distinguish ONC201 from its [4,3-d] linear isomer and supports the angular [3,4-e] structure of ONC201 obtained from both the NCI and Oncoceutics.

IR spectroscopic analysis was also conducted on both ONC201 and the [4,3-d] linear isomer. The IR spectra revealed peaks between the 2000–1000 cm^−1^ (1612, 1490, 1446 cm^−1^), wavelengths that are indicative of the carboxyl, pyridine and amine, and aromatic functional groups, respectively, that are present in both compounds. Peaks within the 2900–3100 cm^−1^ wavelength region indicate the presence of both the aromatic and H-C-N functional groups. Due to the large vibrations occurring at the region below 600 cm^−1^, peaks in these could assigned. An overlay of both spectra demonstrates that ONC201 and the [4,3-d] linear isomer are nearly identical in dichloromethane, supporting the expected conclusion that NMR is better able to distinguish the two isomers than IR (Figure 4C).

### ONC201 reduces cancer cell viability, inactivates Akt and ERK, and induces TRAIL unlike the [4,3-d] linear isomer

**Figure 5:**
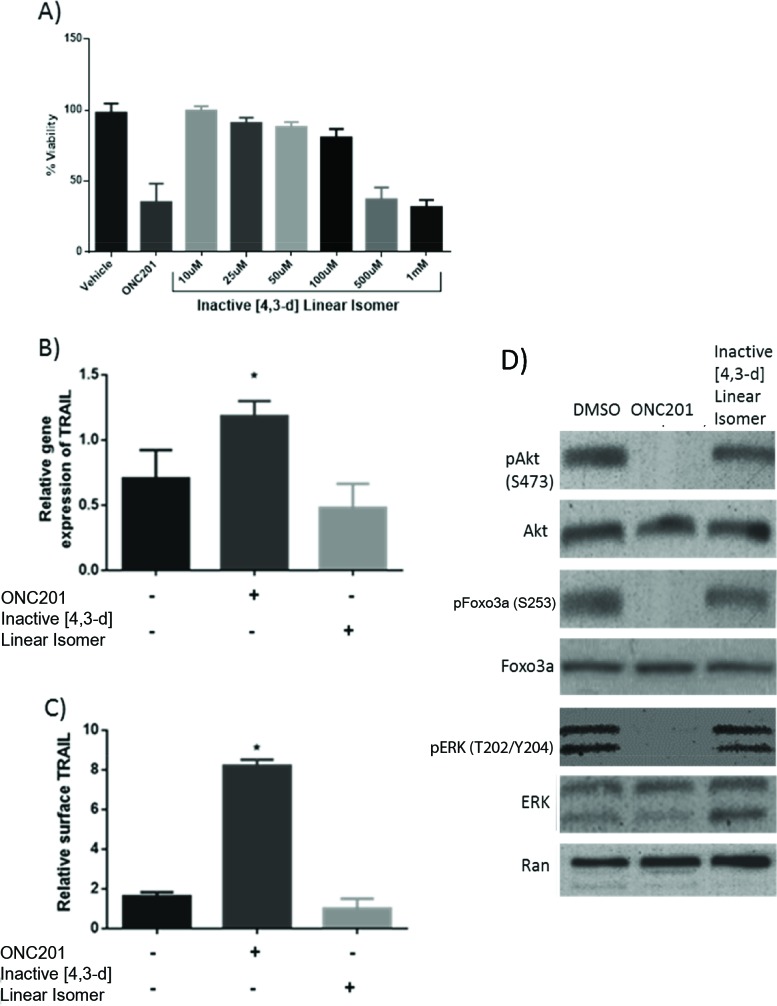
ONC201 induces TRAIL and decreases human colon cancer cell viability at a significantly lower concentration than the linear isomer **(A)** Cell viability in HCT116 cells shows that the [4,3-d] linear isomer compound does not reach a Cells where treated for 72 hours. N=3. **(B)** RT-qPCR analysis of relative TRAIL gene expression normalized to GAPDH and **(C)** TRAIL surface expression in HCT116. Cells were treated with DMSO or 10uM ONC201 or the [4,3-d] linear isomer for 72 hours. N=3. **(D)** Western blot analysis downstream signaling in HCT116 cells treated with DMSO or 10uM ONC201 or the [4,3-d] linear isomer for 72 hours. Phosphorylation sites for Ab: pAkt (S473), pFoxo3a (S253), pERK (T202/Y204). *p < .05 Student's two tailed *t*-test relative to control.

We previously reported that ONC201 induces TRAIL and cell death in the HCT116 colorectal cancer cell line. To compare the biological activity of ONC201 and the [4,3-d] linear isomer, cell viability assays were conducted in HCT116 cells. In stark contrast to ONC201, the [4,3-d] linear isomer was unable to induce cell death below 1 mM, a high dose that is unlikely to be achieved *in vivo* (Figure 5A). The [4,3-d] linear isomer was unable to induce TRAIL in HCT116 cells at any dose tested. ONC201 induced TRAIL gene expression at the RNA and protein level to a similar magnitude as previously reported by Allen *et al* using the NCI material (Figure 5B–5C). Dephosphorylation of Foxo3a, Akt, and ERK was comparable to previously reported results associated with the unique signaling mechanism for ONC201 (Figure 5D) [4]. Thus, the conclusions drawn from Allen *et al*. regarding the preclinical profile of ONC201, including its ability to induce TRAIL, are associated with the angular structure that is ONC201, as distributed by the NCI and Oncoceutics.

## DISCUSSION

We previously reported the identification of ONC201 as an efficacious anti-tumor agent that induces TRAIL pathway activation driven by an upstream indirect dual blockade of Akt and ERK signaling [4]. With the clinical introduction of ONC201, the compound has gained significant attention due to its ideal safety and efficacy drug profile as a small molecule. While our work was in progress to determine the structural differences between the robustly active ONC201 sourced from the NCI and Oncoceutics compared to the inactive commercially sourced compound, Janda reported an alternative angular [3,4-e] structure of TIC10/ONC201 and showed that the linear [4,3-d] structure is inactive, which explained our unpublished observations.

The depiction of the ONC201 structure in 1973 as the [4,3-d] isomer was likely due to an incorrect prediction of the structure of intermediates involved in its synthesis. This structural mis-assignment was likely propagated for decades due to the high degree of similarity between the previously depicted structure and the actual structure. Since both structures have a high degree of structural and electronic similarity and the 1973 synthetic route yields a highly pure product, this issue only recently emerged when an alternative synthetic route for the linear isomer rather than the published synthetic route was used and yielded an inactive product. Furthermore, the mass spectrometry analysis previously reported by *Allen et al*. was consistent with the public domain structure and the disclosed structure from Stähle *et al*.

The detailed structural analyses of ONC201 and the [4,3-d] linear isomer prepared as a reference indicate that the active compound ONC201 is indeed the angular compound represented by the angular [3,4-e] chemical structure. The [4,3-d] linear isomer does not induce cell death or affect the proliferation of cancer cells in a pharmacologically acceptable dose range, suggesting limited therapeutic potential for TRAIL induction for that compound and highlighting the uniqueness of ONC201. Earlier refinement of the structure of ONC201 would not have impacted its discovery as a promising drug substance given the phenotypic screen from a library of publicly available compounds that led to its identification. Nor would the knowledge of structural detail as presented by Jacob *et al* [8] have changed the course of the downstream development of ONC201, though structural elucidation may however help to inform on certain characteristics of the drug. Despite the propagated structural mis-assignment of ONC201, the prior preclinical studies are valid and the therapeutic potential of ONC201 to address unmet oncology needs remains strong.

The publication by Jacob *et al* suggested that their structural findings cast significant doubt on the clinical development of ONC201 due to uncertainty of the actual chemical entity under development. The studies presented herein confirm that the clinical ONC201 dihydrochloride salt material indeed possesses an angular [3,4-e] structure that also represents the same structure distributed by the NCI for preclinical studies and was utilized by Oncoceutics for preclinical pharmacology, IND-enabling studies, and upcoming clinical studies. Since ONC201, which has the angular [3,4-e] structure, has always been the compound under development by Oncoceutics and part of the documentation provided to regulatory agencies, the structural clarification does not affect prior studies. The development of ONC201 continues to advance in parallel with these recent findings clarifying the structure of the compound. Several early phase clinical trials with ONC201 have been initiated to confirm its safety and preliminary efficacy profile in advanced cancer patients that appears to be uniquely robust in preclinical models.

## MATERIALS AND METHODS

### Reagents and cell viability assays

All cell lines were obtained from the American Type Culture Collection. ONC201 was obtained from Oncoceutics as the dihydrochloride salt unless noted otherwise. ONC201 was synthesized by Oncoceutics following a modification of the Stähle procedure and was converted to the dihydrochloride salt for biological evaluation and X-ray crystallography. The free base was prepared by neutralization of the salt and extraction. ONC201/NSC-350625 was obtained from the NCI Developmental Therapeutics Program (DTP) repository. The [4,3-d] linear isomer was prepared by the procedure of Jacob *et al*. [8] and also purchased commercially from MedKoo Biosciences, Inc. (Chapel Hill, NC). NMR spectra of the MedKoo correspond to the previously disclosed linear isomer [8]. Adherent cells were analyzed on a Beckman-Coulter Elite Epics cytometer. Cell viability assays were carried out in 96-well, black-walled, clear-bottomed plates with CellTiter-Glo (Promega; Madison, WI) according to the manufacturer's protocols.

### NMR analysis of ONC201

NMR spectra were acquired at 25°C on a Varian Inova 500 MHz spectrometer. Experiments acquired for each sample included 1D ^1^H, 2D DQCOSY, 2D ROESY [mix = 0.3 sec], 2D ^1^H-^13^C gHMQC, 2D ^1^H-^13^C gHMBCAD. For samples in DMSO, chemical shift values were calibrated with reference to the ^1^H and ^13^C resonances of internal residual DMSO at 2.50 ppm and 39.51 ppm respectively. For the samples in chloroform, ^1^H chemical shift values were calibrated with reference to internal residual chloroform at 7.24 ppm, and for ^13^C referencing a 1D 13C spectrum was acquired and internal CDCl_3_ was used as a standard at 77.23 ppm.

### X-ray crystallography

Crystallization was carried out with 50 mg of ONC201 salt that was heated to boiling with 0.5 mL of ethanol. One drop (~50 μL) of water was added and the mixture was heated to dissolve all the solids. The hot solution was filtered through a plug of cotton into a 2 dram vial and the clear solution was capped and allowed to stand and cool undisturbed at room temperature overnight. The sample formed a cluster of large, well-formed crystals that were kept in the solvent for structural analysis. X-ray intensity data were collected on a Bruker APEXII CCD area detector employing graphite-monochromated. Mo-Kα radiation (λ =0.71073 Å) at a temperature of 100K. Preliminary indexing was performed from a series of thirty-six 0.5° rotation frames with exposures of 10 seconds. A total of 1592 frames were collected with a crystal to detector distance of 37.4 mm, rotation widths of 0.5° and exposures of 5 seconds.

Rotation frames were integrated using SAINTi, producing a listing of unaveraged F2 and σ (F2) values which were then passed to the SHELXTLii program package for further processing and structure solution. A total of 36379 reflections were measured over the ranges 1.74 ≤ θ ≤ 25.40°, −14 ≤ *h* ≤ 14, −12 ≤ *k* ≤ 12, −26 ≤ *l* ≤ 26 yielding 4928 unique reflections (Rint = 0.0151).

The intensity data were corrected for Lorentz and polarization effects and for absorption using SADABSiii (minimum and maximum transmission 0.6888, 0.7452). The structure was solved by direct methods (SHELXS-97iv). The asymmetric unit consists of the dihydrochloride of the parent compound plus a molecule of ethanol solvent. There was an additional region of disordered solvent, which was attributed to water, for which a reliable disorder model could not be devised; the X-ray data were corrected for the presence of disordered solvent using SQUEEZE.

Refinement was by full-matrix least squares based on F2 using SHELXL-97. All reflections were used during refinement. The weighting scheme used was *w* = 1/[σ2 (Fo2) + (0.0378P)2 + 1.3664P] where *P* = (Fo2 + 2Fc2 )/3. Non-hydrogen atoms were refined anisotropically and hydrogen atoms were refined using a riding model. Refinement converged to R1=0.0289 and wR2=0.0772 for 4664 observed reflections for which *F* > 4 σ (F) and R1=0.0302 and wR2=0.0779 and GOF =1.066 for all 4928 unique, non-zero reflections and 311 variables. The maximum Δ/σ in the final cycle of least squares was 0.000 and the two most prominent peaks in the final difference Fourier were +0.304 and −0.246 e/Å3.

### CID mass spectrometry, ^1^H ^13^C NMR, and IR analysis of ONC201 material from NCI

Mass spectral data were obtained using a Varian 500-Qtrap mass spectrometer with an electron spray ion (ESI) source in negative ion mode. The samples were diluted 1:100 in 80:20 acetonitrile:1% formic acid and infused into the source chamber at a flow rate of 10 μL min^−1^ with the following parameters: spray chamber temperature, 0°C; needle voltage, –5500 V; collision energy, 10 eV; declustering potential, 75 V; current gas flow, 40 LFM. Masses of product ions were measured with a micrOTOF QII Q-TOF mass spectrometer (Bruker Co., USA) equipped with an ESI source. The collision energy of the CID for the selected ions was set as standard, with argon as the collision gas. The data were analyzed using the Data Analysis version 4 software package delivered by Bruker Daltonics. NMR experiments were conducted using standard protocols on Bruker Advanced 500 and 600 MHz spectrometers equipped with cold probes. IR experiments were analyzed on a Jasco FT/IR-4100 in dichloromethane solvent.

### TRAIL protein and mRNA expression

For surface TRAIL protein experiments, adherent cells were harvested by brief trypsinization, fixed in 4% paraformaldehyde in phosphate-buffered saline (PBS) for 20 min., incubated with an anti-TRAIL antibody (Abcam, ab2435) at 1:250 overnight, washed and incubated with anti-rabbit Alexa Fluor 488 (Invitrogen) for 30 min., and analyzed as previously described [4].

Primers for TRAIL gene expression (5′- TGC GTG CTG ATC GTG ATC TTC -3′; 5′- GCT CGT TGG TAA AGT ACA CGT A -3′) were tested in HCT116 cells to ensure that the proper size of fragments was generated. GAPDH primer was obtained from PE Applied Biosystems. Total RNA was isolated using Quick-RNA MiniPrep (Zymo Research) and 1 μg was used for reverse transcription and amplification using the SuperScript III First-Strand Synthesis System (Life Technologies) according to the manufacturer's protocol. A master mix of SYBR Green (Life Technologies) and 1 μg of cDNA was used in the PCR reaction. Each tube contained both a gene probe and primers and a GAPDH control probe and primer. Each sample was performed in quadruplicate. A control run without reverse transcriptase yielded no expression, indicating that the samples were free from genomic DNA contamination. Gene amounts were quantitated using the standard curve method of expression relative to GAPDH. Reactions were carried out in 96-well plates using Bio-Rad CFX96 Real Time System.

### Western blot analysis

Western blot analysis used extracts prepared with a buffer of 25 mM Tris-HCL (pH 7.6), 150 mM NaCl, 1% NP-40, 1% sodium deoxycholate, 0.1% SDS with fresh protease inhibitor (Roche). Western blots were completed with NuPage 4% to 12% bis-tris gels and visualized with SuperSignal West Femto (Thermo Scientific) and Odyssey infrared imaging.

### Statistical analysis

For pairwise comparisons, we analyzed the data using the GraphPad two-tailed *t* test (http://www.graphpad.com/quickcalcs/ttest1.cfm).

## SUPPLEMENTARY FIGURES AND TABLES


