# Supplementary material for: The angular structure of ONC201, a TRAIL pathway-inducing compound, determines its potent anti-cancer activity
Source: Oncotarget. 2015 Jan 13;5(24):12728–37. doi: 10.18632/oncotarget.2890 (PMC4350349; doi:10.18632/oncotarget.2890)
Supplement: Supplementary file 1 [file oncotarget-05-12728-s001.pdf]

SUPPLEMENTARY FIGURES AND TABLES

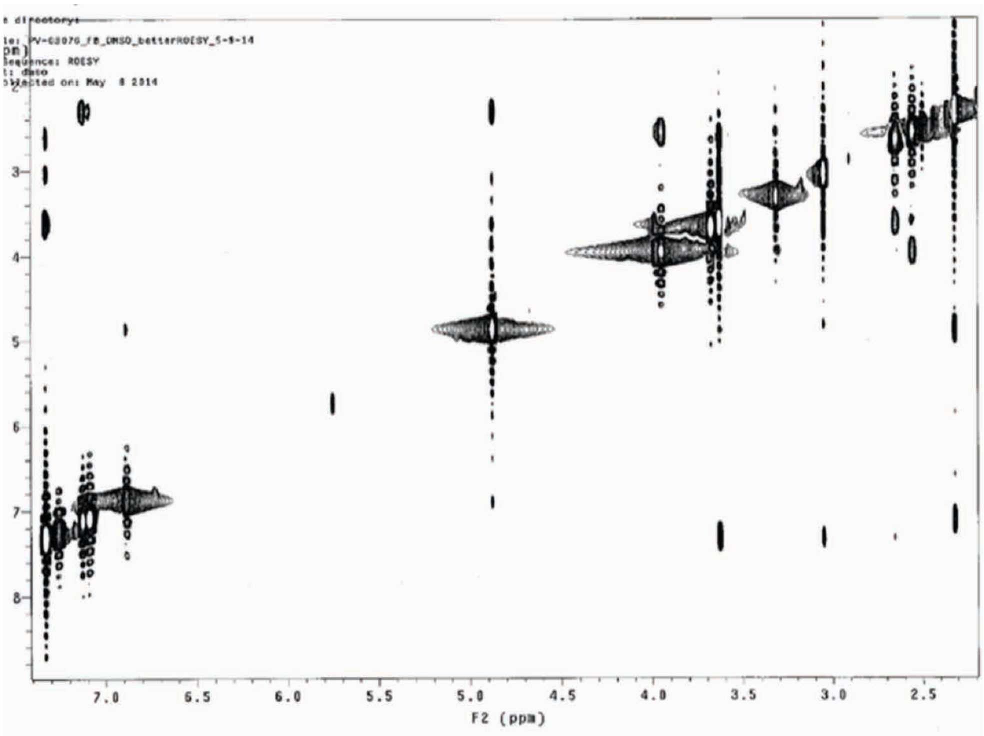

Supplementary Figure S1: 2D ^{1}H-^{1}H ROESY spectrum of ONC201 in d_6-DMSO.

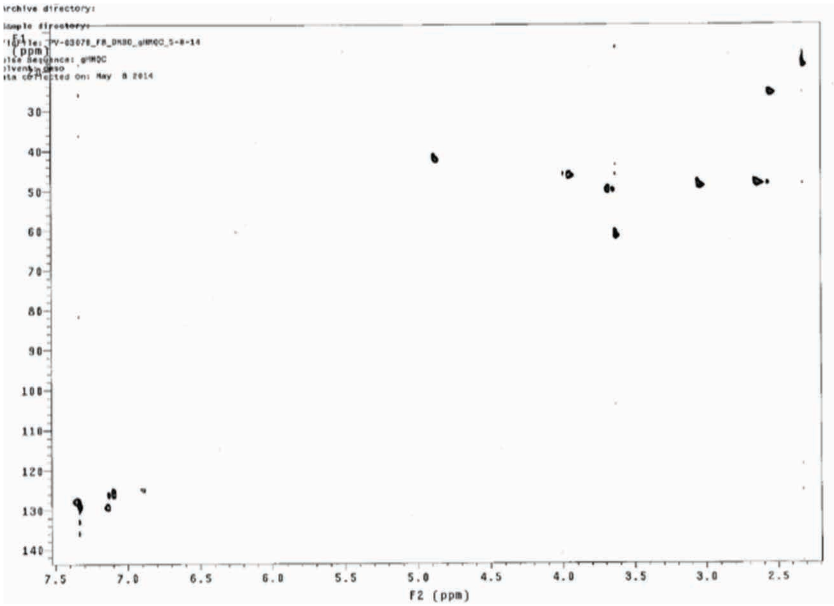

Supplementary Figure S2: 2D ^{1}H-^{13}C gHMQC spectrum of ONC201 in d_6-DMSO.

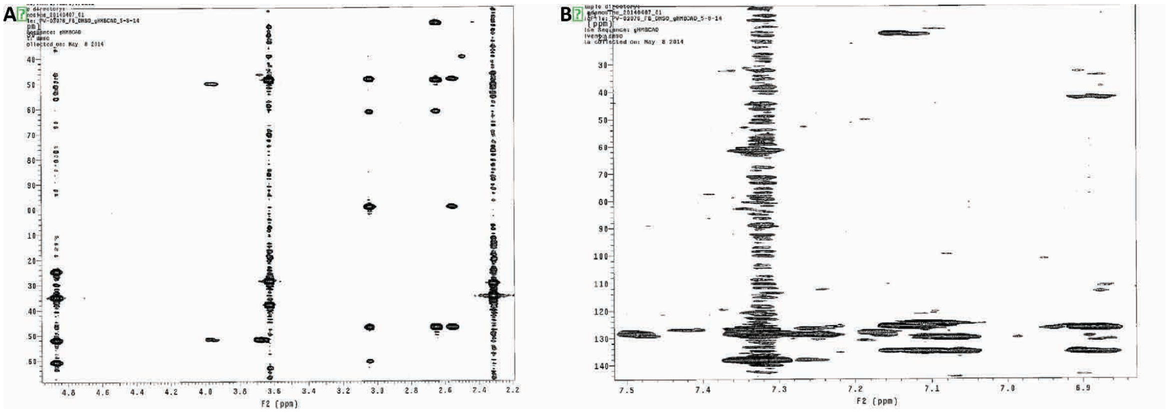

Supplementary Figure S3: **(A)** Upfield and **(B)** downfield region of 2D ^{1}H-^{13}C gHMBCAD spectrum of ONC201 in d_6-DMSO.

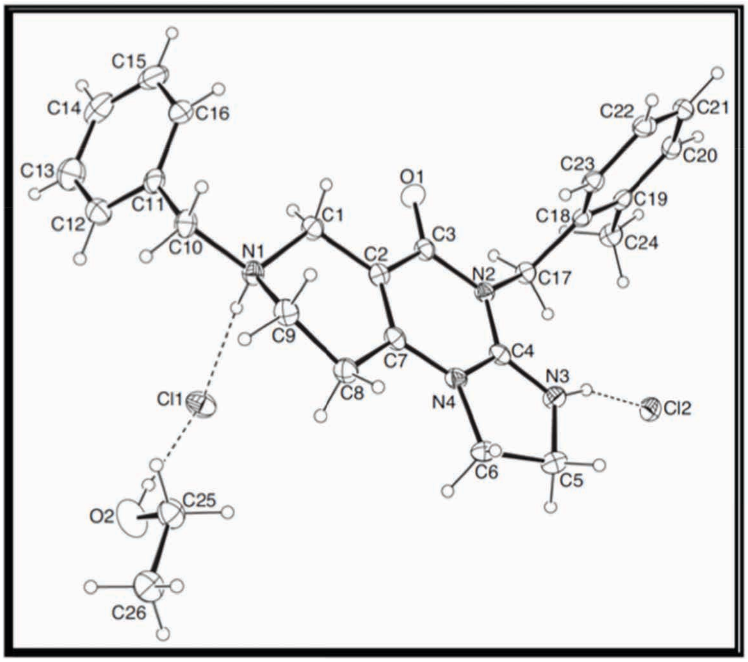

Supplementary Figure S4: ORTEP drawing of ONC201 dihydrochloride structure with 50% probability thermal ellipsoids.

Supplementary Table S1: Summary of X-Ray Structure determination of ONC201 dihydrochloride structure.

Empirical formula	$C_{26}H_{34}N_4O_2Cl_2$
Formula weight	505.47
Temperature	100(1) K
Wavelength	0.71073 Å
Crystal system	monoclinic
Space group	$P2_1/c$
Cell constants:	
a	11.7746(3) Å
b	10.3998(2) Å
c	21.9517(5) Å
β	95.1950(10)°
Volume	2677.02(10) $Å^3$
Z	4
Density (calculated)	1.254 Mg/m^3
Absorption coefficient	0.272 mm^{-1}
F(000)	1072
Crystal size	0.32 x 0.24 x 0.06 mm^3
Theta range for data collection	1.74 to 25.40°
Index ranges	$-14 \le h \le 14$, $-12 \le k \le 12$, $-26 \le l \le 26$
Reflections collected	36379
Independent reflections	4928 [R(int) = 0.0151]
Completeness to theta = 25.40°	99.9 %
Absorption correction	Semi-empirical from equivalents
Max. and min. transmission	0.7452 and 0.6888
Refinement method	Full-matrix least-squares on F^2
Data / restraints / parameters	4928 / 0 / 311
Goodness-of-fit on F^2	1.066
Final R indices [I>2sigma(I)]	R1 = 0.0289, wR2 = 0.0772
R indices (all data)	R1 = 0.0302, wR2 = 0.0779
Largest diff. peak and hole	0.304 and -0.246 e.$Å^{-3}$

Supplementary Table S2: Refined positional parameters for ONC201 dihydrochloride structure, excluding hydrogens.

Atom	x	y	z	U_{eq}, $Å^2$
C1	0.24400(11)	0.95070(12)	0.14757(5)	0.0172(3)
C2	0.28090(10)	0.82303(12)	0.17399(5)	0.0157(2)
C3	0.32697(10)	0.73340(12)	0.13249(5)	0.0157(2)
C4	0.34884(10)	0.58406(12)	0.21644(5)	0.0155(2)
C5	0.35291(12)	0.48312(13)	0.30893(6)	0.0218(3)
C6	0.29742(11)	0.61564(13)	0.31441(6)	0.0210(3)
C7	0.27053(10)	0.79035(12)	0.23279(6)	0.0160(2)
C8	0.22097(11)	0.87906(12)	0.27691(6)	0.0188(3)
C9	0.20800(11)	1.01479(12)	0.25157(5)	0.0185(3)
C10	0.13315(11)	1.14974(12)	0.16495(6)	0.0203(3)
C11	0.08993(11)	1.15801(12)	0.09839(6)	0.0187(3)
C12	-0.02590(12)	1.15066(14)	0.08038(6)	0.0251(3)
C13	-0.06626(13)	1.15985(15)	0.01895(7)	0.0309(3)
C14	0.01002(14)	1.17297(14)	-0.02461(7)	0.0314(3)
C15	0.12501(14)	1.17927(14)	-0.00740(7)	0.0321(3)
C16	0.16528(12)	1.17367(13)	0.05378(7)	0.0255(3)
C17	0.39945(10)	0.51418(11)	0.11605(5)	0.0152(2)
C18	0.52420(10)	0.52407(12)	0.10534(5)	0.0145(2)
C19	0.56764(10)	0.42974(12)	0.06780(5)	0.0160(2)
C20	0.68077(11)	0.43991(13)	0.05460(5)	0.0188(3)
C21	0.74934(11)	0.54095(13)	0.07701(6)	0.0207(3)
C22	0.70618(11)	0.63290(13)	0.11435(6)	0.0189(3)
C23	0.59365(10)	0.62345(12)	0.12861(5)	0.0163(2)
C24	0.49394(11)	0.32199(12)	0.04178(6)	0.0197(3)
N1	0.16108(9)	1.01341(10)	0.18576(5)	0.0168(2)
N2	0.36042(8)	0.61299(10)	0.15739(5)	0.0148(2)
N3	0.37882(9)	0.47786(10)	0.24497(5)	0.0179(2)
N4	0.30281(9)	0.67061(10)	0.25284(4)	0.0159(2)
O1	0.33734(8)	0.75527(9)	0.07856(4)	0.01955(19)
Cl1	-0.05749(3)	0.85901(3)	0.195197(14)	0.02505(9)
Cl2	0.43555(3)	0.21186(3)	0.199098(14)	0.02173(9)
C25	-0.04631(11)	1.00244(14)	0.35857(6)	0.0247(3)
C26	-0.08679(12)	1.02224(14)	0.42091(6)	0.0254(3)
O2	-0.14117(8)	0.96661(12)	0.31771(5)	0.0333(3)

$U_{eq}=\frac{1}{3}[U_{11}(aa^*)^2+U_{22}(bb^*)^2+U_{33}(cc^*)^2+2U_{12}aa^*bb^*\cos\gamma+2U_{13}aa^*cc^*\cos\beta+2U_{23}bb^*cc^*\cos\alpha]$

Supplementary Table S3: Positional Parameters for ONC201 dihydrochloride hydrogens in structure.

Atom	x	y	z	U_{iso}, A^2
H1a	0.3101	1.0057	0.1457	0.023
H1b	0.2088	0.9389	0.1063	0.023
H5a	0.4219	0.4759	0.3364	0.029
H5b	0.3008	0.4149	0.3179	0.029
H6a	0.2192	0.6077	0.3244	0.028
H6b	0.3398	0.6678	0.3453	0.028
H8a	0.2700	0.8807	0.3149	0.025
H8b	0.1469	0.8470	0.2859	0.025
H9a	0.1572	1.0633	0.2754	0.025
H9b	0.2817	1.0572	0.2552	0.025
H10a	0.0759	1.1846	0.1894	0.027
H10b	0.2011	1.2023	0.1721	0.027
H12	-0.0772	1.1395	0.1097	0.033
H13	-0.1442	1.1571	0.0074	0.041
H14	-0.0163	1.1776	-0.0658	0.042
H15	0.1762	1.1874	-0.0371	0.043
H16	0.2431	1.1804	0.0651	0.034
H17a	0.3543	0.5206	0.0770	0.020
H17b	0.3856	0.4301	0.1330	0.020
H20	0.7110	0.3777	0.0303	0.025
H21	0.8242	0.5469	0.0670	0.027
H22	0.7520	0.7004	0.1298	0.025
H23	0.5647	0.6845	0.1540	0.022
H24a	0.5356	0.2717	0.0147	0.030
H24b	0.4269	0.3567	0.0196	0.030
H24c	0.4721	0.2685	0.0744	0.030
H1	0.0955	0.9667	0.1825	0.022
H3	0.4097	0.4132	0.2284	0.024
H25a	-0.0128	1.0812	0.3447	0.033
H25b	0.0112	0.9355	0.3602	0.033
H26a	-0.1420	1.0903	0.4191	0.038
H26b	-0.0232	1.0446	0.4494	0.038
H26c	-0.1210	0.9444	0.4340	0.038
H2	-0.1190	0.9448	0.2849	0.050

Supplementary Table S4: Refined Thermal Parameters (U's) for ONC201 dihydrochloride structure.

Atom	U_{11}	U_{22}	U_{33}	U_{23}	U_{13}	U_{12}
C1	0.0196(6)	0.0169(6)	0.0155(6)	-0.0019(5)	0.0037(5)	0.0029(5)
C2	0.0144(6)	0.0165(6)	0.0163(6)	-0.0020(5)	0.0010(4)	0.0008(5)
C3	0.0141(6)	0.0157(6)	0.0175(6)	-0.0009(5)	0.0015(4)	-0.0003(5)
C4	0.0116(5)	0.0178(6)	0.0170(6)	-0.0012(5)	0.0013(4)	-0.0022(5)
C5	0.0249(7)	0.0228(7)	0.0181(6)	0.0047(5)	0.0033(5)	0.0000(5)
C6	0.0237(6)	0.0252(7)	0.0148(6)	0.0040(5)	0.0059(5)	0.0011(5)
C7	0.0119(5)	0.0191(6)	0.0167(6)	-0.0024(5)	0.0004(4)	-0.0009(5)
C8	0.0197(6)	0.0216(6)	0.0154(6)	-0.0020(5)	0.0029(5)	0.0023(5)
C9	0.0202(6)	0.0206(6)	0.0147(6)	-0.0046(5)	0.0014(5)	0.0020(5)
C10	0.0222(6)	0.0150(6)	0.0237(7)	-0.0026(5)	0.0015(5)	0.0039(5)
C11	0.0227(6)	0.0118(6)	0.0218(6)	-0.0003(5)	0.0032(5)	0.0036(5)
C12	0.0240(7)	0.0279(7)	0.0239(7)	0.0011(6)	0.0046(5)	0.0032(6)
C13	0.0311(8)	0.0313(8)	0.0290(8)	-0.0002(6)	-0.0040(6)	0.0028(6)
C14	0.0528(10)	0.0204(7)	0.0204(7)	0.0021(5)	0.0004(6)	0.0060(7)
C15	0.0491(9)	0.0207(7)	0.0293(7)	0.0074(6)	0.0184(7)	0.0058(7)
C16	0.0268(7)	0.0177(6)	0.0332(8)	0.0060(6)	0.0100(6)	0.0017(5)
C17	0.0175(6)	0.0128(6)	0.0154(6)	-0.0023(4)	0.0022(5)	0.0009(5)
C18	0.0165(6)	0.0151(6)	0.0120(5)	0.0027(4)	0.0015(4)	0.0019(5)
C19	0.0206(6)	0.0156(6)	0.0117(5)	0.0026(5)	0.0007(5)	0.0030(5)
C20	0.0213(6)	0.0213(6)	0.0140(6)	0.0017(5)	0.0037(5)	0.0077(5)
C21	0.0152(6)	0.0291(7)	0.0179(6)	0.0065(5)	0.0025(5)	0.0021(5)
C22	0.0188(6)	0.0222(6)	0.0154(6)	0.0033(5)	-0.0007(5)	-0.0038(5)
C23	0.0201(6)	0.0157(6)	0.0130(6)	0.0002(5)	0.0017(5)	0.0013(5)
C24	0.0255(7)	0.0152(6)	0.0187(6)	-0.0025(5)	0.0045(5)	0.0023(5)
N1	0.0176(5)	0.0159(5)	0.0168(5)	-0.0020(4)	0.0018(4)	0.0019(4)
N2	0.0152(5)	0.0148(5)	0.0149(5)	-0.0009(4)	0.0035(4)	0.0009(4)
N3	0.0203(5)	0.0164(5)	0.0171(5)	0.0011(4)	0.0019(4)	0.0008(4)
N4	0.0158(5)	0.0192(5)	0.0132(5)	0.0001(4)	0.0029(4)	0.0010(4)
O1	0.0266(5)	0.0181(4)	0.0146(4)	-0.0001(3)	0.0050(4)	0.0035(4)
Cl1	0.02173(17)	0.03089(19)	0.02277(17)	-0.00372(13)	0.00328(12)	-0.00604(13)
Cl2	0.02096(16)	0.01701(16)	0.02741(17)	0.00375(12)	0.00331(12)	0.00240(11)
C25	0.0196(6)	0.0284(7)	0.0258(7)	-0.0009(6)	0.0000(5)	-0.0004(6)
C26	0.0224(7)	0.0270(7)	0.0264(7)	-0.0020(6)	0.0005(5)	-0.0024(6)
O2	0.0235(5)	0.0482(7)	0.0273(5)	-0.0119(5)	-0.0028(4)	0.0054(5)

The form of the anisotropic displacement parameter is:
$\exp[-2\pi(a^{*2}U_{11}h^2+b^{*2}U_{22}k^2+c^{*2}U_{33}l^2+2b^*c^*U_{23}kl+2a^*c^*U_{13}hl+2a^*b^*U_{12}hk)]$

Supplementary Table S5: Bond distances in ONC201 dihydrochloride structure.

C1-N1	1.4932(15)	C1-C2	1.4975(17)	C2-C7	1.3510(17)
C2-C3	1.4431(17)	C3-O1	1.2224(15)	C3-N2	1.4082(16)
C4-N3	1.3026(16)	C4-N4	1.3494(16)	C4-N2	1.3496(16)
C5-N3	1.4645(16)	C5-C6	1.5346(19)	C6-N4	1.4741(15)
C7-N4	1.3633(16)	C7-C8	1.4939(17)	C8-C9	1.5198(18)
C9-N1	1.4994(15)	C10-C11	1.5056(18)	C10-N1	1.5165(16)
C11-C12	1.3872(19)	C11-C16	1.3892(18)	C12-C13	1.392(2)
C13-C14	1.377(2)	C14-C15	1.374(2)	C15-C16	1.384(2)
C17-N2	1.4718(15)	C17-C18	1.5120(16)	C18-C23	1.3864(17)
C18-C19	1.4068(17)	C19-C20	1.3926(18)	C19-C24	1.4984(17)
C20-C21	1.3878(19)	C21-C22	1.3856(19)	C22-C23	1.3924(18)
C25-O2	1.4175(16)	C25-C26	1.5035(19)		

Supplementary Table S6: Bond angles in ONC201 dihydrochloride structure.

N1-C1-C2	110.57(10)	C7-C2-C3	120.82(11)	C7-C2-C1	123.02(11)
C3-C2-C1	116.14(10)	O1-C3-N2	119.50(11)	O1-C3-C2	124.57(11)
N2-C3-C2	115.93(10)	N3-C4-N4	112.75(11)	N3-C4-N2	127.18(11)
N4-C4-N2	120.07(11)	N3-C5-C6	103.64(10)	N4-C6-C5	102.85(10)
C2-C7-N4	119.44(11)	C2-C7-C8	122.58(11)	N4-C7-C8	117.95(11)
C7-C8-C9	111.62(10)	N1-C9-C8	111.09(10)	C11-C10-N1	113.00(10)
C12-C11-C16	118.67(12)	C12-C11-C10	120.70(12)	C16-C11-C10	120.62(12)
C11-C12-C13	120.85(13)	C14-C13-C12	119.52(14)	C15-C14-C13	120.17(14)
C14-C15-C16	120.48(13)	C15-C16-C11	120.28(14)	N2-C17-C18	114.23(10)
C23-C18-C19	120.14(11)	C23-C18-C17	122.84(11)	C19-C18-C17	116.97(11)
C20-C19-C18	118.31(11)	C20-C19-C24	120.61(11)	C18-C19-C24	121.07(11)
C21-C20-C19	121.36(12)	C22-C21-C20	119.97(12)	C21-C22-C23	119.45(12)
C18-C23-C22	120.75(11)	C1-N1-C9	110.03(9)	C1-N1-C10	111.98(10)
C9-N1-C10	109.40(9)	C4-N2-C3	121.47(10)	C4-N2-C17	120.06(10)
C3-N2-C17	118.27(10)	C4-N3-C5	111.00(10)	C4-N4-C7	122.23(10)
C4-N4-C6	109.70(10)	C7-N4-C6	128.05(10)	O2-C25-C26	108.38(11)
